# Chang-wei-qing, a Chinese herbal formula, ameliorates colitis-associated tumour development via inhibiting NF-κB and STAT3 signalling pathway

**DOI:** 10.1080/13880209.2019.1577465

**Published:** 2019-03-24

**Authors:** Guangsheng Wan, Manli Xie, Xinyan Zhang, Meiying Li

**Affiliations:** a Oncology Department of traditional Chinese Medicine, Shanghai University of Traditional Chinese Medicine Affiliated PUTUO Hospital, Shanghai, China;; b The Obstetrics & Gynecology Hospital of Fudan University, Shanghai, China;; c Department of Ultrasound, Shanghai University of Traditional Chinese Medicine Affiliated PUTUO Hospital, Shanghai, China

**Keywords:** Colitis-associated cancer, β-glucuronidase, pro-inflammatory response

## Abstract

**Context:** Chang-wei-qing (CWQ) is a Chinese herbal recipe with clinical efficacy. However, the molecular mechanism underlying its recognized therapeutic benefits against colorectal cancer is still elusive.

**Objective:** To investigate the potential beneficial effects of CWQ in drug-induced colitis-associated cancer (CAC) model and its mechanistic involvements in this disease.

**Materials and methods:** Colitis-associated cancer model was induced by azoxymethane (AOM) and dextran sulphate sodium (DSS). CWQ was administrated by gavage. Colon length and tumour size were determined after resection. The colitis was systematically scored. The microbiota and population of *Faecalibacterium prausnitzii (F. prausnitzii)* Hauduroy & Duncan was analysed by quantitative polymerase chain reaction (PCR). β-Glucuronidase, d-lactose and endotoxin were determined with commercially available kits. Pro-inflammatory cytokines were analysed in the colon tissues. Relative protein expressions were determined by Western blotting.

**Results:** High concentration CWQ significantly restored the colon length, decreased tumour number and size (1.7 ± 0.6 vs. 2.8 ± 0.4 mm, *p* < 0.01) and reduced colitis score (11.8 ± 2.1 vs. 18.2 ± 2.3, *p* < 0.01). CWQ also suppressed expansion of *F. prausnitzii* population (0.029 ± 0.015% vs. 0.052 ± 0.019%, *p* < 0.01). CWQ greatly inhibited the activity of β-glucuronidase and leakage of d-lactose and endotoxin. Meanwhile, the pro-inflammatory cytokines were remarkably decreased in CAC mice in response to CWQ treatment. We further demonstrated that CWQ inhibited both NF-κB and STAT3 signalling.

**Conclusions:** We for the first time demonstrated the antitumour properties of CWQ *in vivo* via inhibiting NF-κB and STAT3 signalling.

## Introduction

Colorectal cancer is one of the most common human malignancies derived from the digestive tract. In 2012, 1.4 million new cases were diagnosed and 69,400 deaths were claimed by this disease (Siegel et al. 2013). The clinical options available for colorectal cancer include some combination of surgery, radiotherapy, chemotherapy and targeted therapy (Ahmed et al. 2014). Surgery is applicable to colorectal cancer patients at early stage for curative purpose, while chemotherapy is standard treatment in combination with either surgery or radiotherapy depending on the stage of this disease. Bone marrow cell therapy has been reported to exhibit tumour suppressive effects in rat colon cancer model (El-Khadragy et al. 2018). The prognosis of this disease was relatively favourable and 65% 5-year survival rate was reported in the USA. A number of risk factors have been characterized to associate with etiology of colorectal cancer, and most cases are related to old age and lifestyle (Simon 2016). Genetic aberrance also contributes to the minority of incidence (Markowitz and Bertagnolli 2009). Some other factors, such as diet, obesity, smoking, and lack of physical activity, were reported to be linked to the occurrence of colorectal cancer (Peeters et al. 2015). Notably, chronic inflammatory bowel diseases, such as Crohn’s disease and ulcerative colitis, pose as increased risk factors of colon cancer (Kim and Chang 2014). For the high-risk population with inflammatory bowel diseases, both prevention with aspirin and regular colonoscopies are recommended.

Microbial dysbiosis has received intensive interest from the research community with respect of its potential link to the etiology of colon cancer (Sheflin et al. 2014). Dysbacteriosis fundamentally changes the symbiotic relationship between host and gut microbiota and promotes disease occurrence and development (Candela et al. 2014). Until now, several bacterial species have been characterized with putative carcinogenetic properties during the incidence of colon cancer, such as *Faecalibacterium prausnitzii (F. prausnitzii)* and *Streptococcus bovis* (Sobhani et al. 2011). Multiple mechanisms have been proposed and demonstrated underlying the oncogenic features of microbial dysbiosis, such as genotoxicity, virulence factors, pro-inflammation, host defence impairment, toxic metabolites and oxidative microenvironment.

Chang-wei-qing (CWQ) is a Chinese herbal formula proposed by the famous traditional Chinese medicine practitioner Fan ZhongZe with proven clinical efficacy. CWQ is widely prescribed in the traditional Chinese medicine hospitals for auxiliary treatment of alimentary canal malignancies. The confirmative therapeutic effects of CWQ have been documented in reduction of the post-surgery recurrence and metastasis, remission of toxicity and side effects of chemotherapy, improvement in drug resistance and extension of overall survival. Despite of the evidence-based therapeutic effects, insightful investigations into molecular mechanisms are still rare. Xu et al. (2010) first reported the effect of CWQ on nuclear translocation of Y-box binding protein-a and expression of P-glycoprotein in human colon cancer cell line with drug-resistance induced by vincristine (Xu et al. 2010). Li et al. (2011) proposed the *in vitro* anti-metastatic effect of CWQ through anti-invasion of hypoxic colorectal carcinoma LoVo cells (Li et al. 2011). Zhang et al. (2012, 2015) investigated the effect of medicated serum prepared with CWQ on the pharmacokinetics of oxaliplatin in colon cancer cells and subsequently demonstrated that CWQ synergistically enhanced antitumour activity of oxaliplatin (Zhang et al. 2012, 2015). It is noteworthy that all of the abovementioned studies were performed *in vitro* with cell culture, which might not fully recapitulate the disease progression and responses. Therefore, here we employed drug-induced colitis-associated cancer (CAC) model to investigate the potential beneficial effects of CWQ, and attempted to understand its underlying molecular mechanism.

## Materials and methods

### Animal model

The C57BL/6 mice were purchased from Biocytogen (Beijing, China) and quarantined for one week before experiments. All animals were housed in specific pathogen-free environment. Autoclaved food and drinking water were supplied *ad libitum*. The animal-related study was performed in strict accordance with the National Institute of Health guideline and regulation. The experimental protocol was approved by the Ethics Committee of Shanghai University of Traditional Chinese Medicine Affiliated Putuo Hospital. CAC was induced as previously described (Grivennikov et al. 2009). Briefly, C57BL/6 mice were injected intraperitoneally (i.p.) with 10 mg/kg azoxymethane (AOM; Sigma, St. Louis, MO), and after 5 days received drinking water containing 2.5% dextran sulphate sodium (DSS, Sigma, St. Louis, MO) for 2 days. Mice were then given regular drinking water for 14 days, followed by two additional DSS treatment cycles at day 25–30 and day 45–50. Colons were removed on day 75, flushed with ice-cold phosphate-buffered saline (PBS), and tumours were counted. Macroscopic tumours were measured with calipers. The extent of inflammation was measured and scored as described.

### Preparation of CWQ

All crude drugs of CWQ were purchased from a local herbal medicine market. Components have been identified and provided by the Department of Pharmacy of Putuo Hospital, Shanghai University of Traditional Chinese Medicine. Briefly, *Astragalus membranaceus* (Fisch.) Bunge (Leguminosae) (dried root), *Atractylodes macrocephala* Koidz. (Asteraceae) (dried root), *Codonopsis pilosula* (Franch.) Nannf. (Campanulaceae) (dried root), *Akebia quinata* (Thunb.) Decne. (Lardizabalaceae) (fruit), *Polyporus umbellatus* (Pers.) Fr. (Polyporaceae) (dried root), *Coix lacryma-jobi* L. var*. mayuen* (Roman.) Stapf (Poaceae) (seeds), *Vitis quinquangularis* Rehder (Vitaceae) (dried root) and *Sargentodoxa cuneata* (Oliv.) Rehder & E.H.Wilson (Sargentodoxacea) (stem) were mixed at a ratio of 10:5:5:8:8:10:10:10. The herbal mixture was decocted twice with 3000 mL water for 1 h each time, and the decoction was filtrated and stored at 4 °C for further use. Decoction liquid was prepared for animal administration at 1.33 g/mL. The mice were randomly divided into five groups (*n* = 6 per group): non-treatment (NT), AOM/DSS treatment (AOM/DSS), Bifico (Bif) treatment (AOM/DSS/Bif), low dose of CWQ (AOM/DSS/CWQL) and high dose of CWQ (AOM/DSS/CWQH). The mice in the low dose of CWQ were given CWQ 5 mg/kg by gavage 2 weeks before the beginning of the experiment each day until the end of the experiment. The mice in the high dose of CWQ were given CWQ 10 mg/kg by gavage 2 weeks before the beginning of the experiment each day until the end of the experiment. The mice in the Bif group were given Bif capsules (4.2 g/kg, dissolved in 200 mL physiological saline) by gavage 2 weeks before the beginning of the experiment each day until the end of the experiment.

### Microbiota analysis

Microbiota analysis was described previously (Klimesova et al. 2013). Briefly, total DNA of stool samples were isolated using ZR Fecal DNA Kit (Zymo Research, ‎Irvine, CA) according to the manufacturer’s instruction. The isolated DNA was stored at –20 °C for further analyses. We performed quantitative polymerase chain reaction (qPCR) with specific primers to determine the numbers of the total bacteria (*Eubacteria*), and *F. prausnitzii* in the stool samples. The primers were as follows: All Eubacteria 5′-TCCTACGGGAGGCAGCAGT-3′ (forward) and 5′-GGACTACCAGGGTATCTATCCTGTT-3′ (reverse). *F. prausnitzii* 5′-GATGGCCTCGCGTCCGATTAG-3′ (forward) and 5′-CCGAAGACCTTCTTCCTCC-3′ (reverse). The qPCR 2× SYBR Master mix (Top-Bio, Vestec, Czech Republic) was used along with Stratagene mx3005P (Agilent Technologies, ‎Santa Clara, CA) equipment. Three-log diluted DNA isolated from known number of cells was used as standard for absolute quantification.

### Histopathology examination

The colon was resected at the endpoint of experiments and inspected macroscopically for the potential pathological lesions. The histopathological evaluation was performed by three independent experienced pathologists with conventional criteria, and normal mucosa, dysplasia and carcinoma were determined.

### β-Glucuronidase determination

β-Glucuronidase was measured as described previously (Reynoso-Camacho et al. 2015). Briefly, enzyme was extracted from lyophilized and weighed stool pellets into 1 mL of acetate buffer (50 mM; pH 7) and incubated for 2 h at 4 °C. Fifty microlitres of extract was added into 100 μL of acetate buffer (50 mM; pH 5) with 50 μL of 2.5 mM MUG substrate (4-methylumbelliferyl-β-d**-**glucuronide, Warrington, UK) and incubated at 37 °C. Product fluorescence was measured at the beginning and after 2 h on microplate reader (Tecan, Männedorf, Switzerland) using 388 nm as excitation and 480 nm as emission wavelength.

### Serum d-lactose and endotoxin determination

The method to determine serum **d-**lactose and endotoxin was referred to the established protocol (Nagy et al. 2002). The peripheral blood samples were collected from CAC model mice. The serum d-lactose and endotoxin were measured using the d-lactose (Jiancheng Bioengineering, Nanjing, China) and endotoxin (Bioendo Technology, Xiamen, China) test kits following the manufacturer’s instruction.

### Enzyme-linked immunosorbent assay (ELISA)

The protein lysates from the homogenates of CAC tissues were prepared in radioimmunoprecipitation assay (RIPA) buffer. The pro-inflammatory response was evaluated by the ELISA method. The commercially available ELISA kits (IL-6: ab100712; IL-1β: ab100704; IL-17: ab100702; TNF-α: ab208348; IFN-γ: ab46081) were used following the manufacturer’s instruction.

### Western blotting

Western blot analysis was conducted as previously reported (Lin et al. 2011). Total protein was extracted in RIPA lysis buffer from the indicated CAC tissue samples by homogenizer on ice. Cell debris was completely discarded via refrigerated centrifugation. Protein concentration was determined by the standard samples using the BCA method (ThermoFisher, Waltham, MA). Protein was resolved by sodium dodecyl sulphate polyacrylamide gel electrophoresis and transferred onto polyvinylidene difluoride membrane on ice. Incubation with primary antibodies was performed at 4 °C overnight after blocking with 5% skim milk to eliminate nonspecific binding and background signal. The target protein was blotted by horseradish peroxidase-conjugated secondary antibody and visualized with the enhanced chemiluminescent kit (Millipore, Billerica, MA). The semi-quantitative analysis was performed using densitometry.

### Statistical analysis

All results were obtained from at least three independent biological repeats. Data processing was performed with GraphPad PRISM 7.0 software (San Diego, CA). The one-way ANOVA followed by Turkey’s test was employed for statistical comparison. The significance *p* value was calculated and *p* < 0.05 was considered as significantly different.

## Results

### CWQ ameliorates colitis-associated tumour development in mouse model

**Figure 1.**
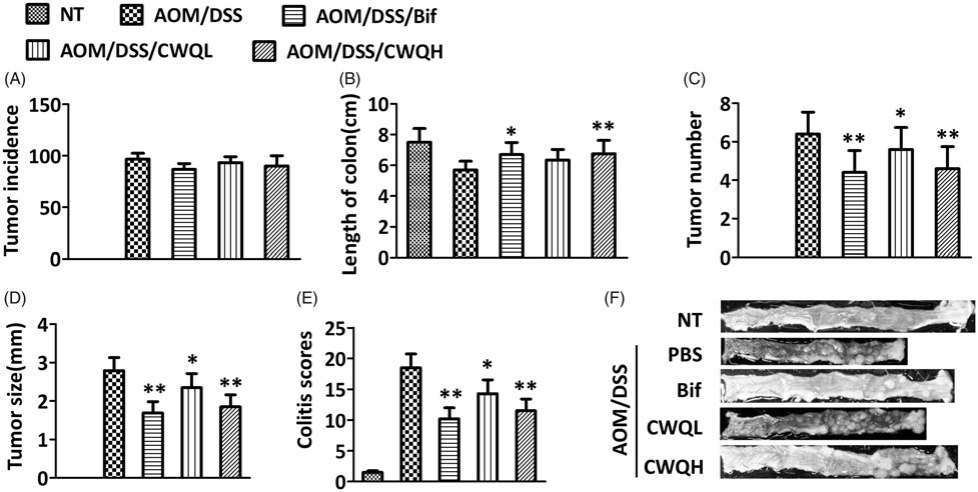
Chang-wei-qing ameliorated colitis-associated tumour development in mouse model. (A) Tumour incidence. (B) Length of colon. (C) Tumour number. (D) Tumour size. (E) Colitis severity scores. (F) Representative histopathological images. NT: none treated, control group; AOM/DSS: treated with AOM, DSS and PBS, colitis-associated cancer group; AOM/DSS/Bif: treated with AOM, DSS and Bifico; AOM/DSS/CWQL: treated with AOM, DSS and low dose Chang-wei-qing; AOM/DSS/CWQH: treated with AOM, DSS and high dose Chang-wei-qing. Data are means ± SD (*n* = 6 for each group). Experiments were repeated for three times. *p < 0.05, **p < 0.01 vs. AOM/DSS group.

To investigate the potential beneficial effects of CWQ on the CAC, here we first set out to establish CAC mouse model. The successful induction of colitis-associated colorectal cancer was histologically confirmed at the endpoint of experiment. For comparison purpose, we employed Bif in our study in view of its probiotic properties. As shown in Figure 1(A), 100% incidence of colorectal tumour was achieved in our protocol, and no significant difference was observed in both Bif and CWQ groups in comparison with vehicle control. The length of colon, which was greatly shortened in the CAC mice, was significantly restored in response to Bif treatment. Likewise, high dose of CWQ demonstrated the comparable effect on the colon length, whereas low dose of CWQ exerted negligible influence (Figure 1(B)). In addition, the tumour number and average tumour size was also monitored in response to either Bif or CWQ treatment. As shown in Figure 1(C), the total tumour number was remarkably decreased by either Bif or CWQ treatment in comparison with vehicle group. Notably, high dose of CWQ exhibited higher suppressive capacity against tumour growth. The similar phenotype was observed with respect to the average tumour size (Figure 1(D)). Therefore, our data suggested that high dose of CWQ displayed comparable antitumour activities and significantly ameliorated colitis-associated tumour development. Of note, inflammation was evaluated at the endpoint of our experiment, where both Bif and CWQ greatly mitigated the colitis induced by chemical challenge, which implicated that both Bif and CWQ might play antitumour roles via resolution of colitis (Figure 1(E)). The representative histological images for each group are also presented in Figure 1(F).

### CWQ ameliorates microbiota changes in CAC mouse model

**Figure 2.**
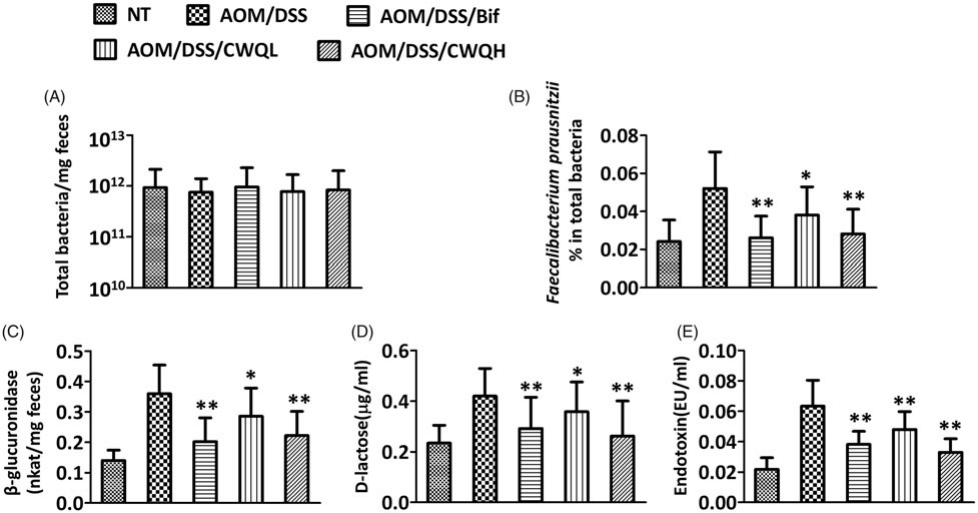
Chang-wei-qing ameliorated microbiota changes in CAC mouse model. (A) The total count of bacteria. (B) The relative numbers of *Faecalibacterium prausnitzii*. Faecal microbiota was assessed by quantitative PCR using specific primers. (C) Activity of β-glucuronidase was measured in the colon content. (D) d-Lactose and (E) endotoxin were measured in the peripheral blood. Data are means ± SD (*n* = 6 for each group). Experiments were repeated for three times. *p < 0.05, **p < 0.01 vs. AOM/DSS group.

Next, we sought to analyse the possible influence of CWQ on the microbiota in the CAC model mice. The total amount of bacteria was constant across different groups in spite of either Bif or CWQ treatments (Figure 2(A)), which indicated that neither Bif nor CWQ impacted the abundance of microbiota. However, the relative number of *F. prausnitzii*, which was quantified by q-PCR, was significantly increased in the CAC mouse model and nearly completely restored by either Bif or high dose of CWQ (Figure 2(B)). Low dose of CWQ partially suppressed the abundance of *F. prausnitzii*, which was consistent with our previous observations with respect to the antitumour activity. The bacteria-related β-glucuronidase was also measured in the CAC mice in response to either Bif or CWQ treatment. The relative content of β-glucuronidase was significantly induced in the colitis disease model, which was further suppressed by Bif or CWQ treatment (Figure 2(C)). In addition, the leakage of d-lactose and endotoxin into peripheral blood, which reflected the integrity of intestinal mucosa, was further determined using commercial test kits. In the CAC model mice, both d-lactose and endotoxin were significantly higher than normal control, which unambiguously suggested severe impairment of intestinal mucosa. Applications of either Bif or CWQ tremendously lowered the serous levels of both d-lactose and endotoxin, which suggested the evidently protective actions of these drugs on integrity of intestinal mucosa (Figure 2(D,E)). In the same manner, low dose of CWQ demonstrated moderate effects in comparison with high dose. In summary, our data demonstrated that CWQ influenced the microbiota composition, inhibited β-glucuronidase activity and improved the integrity of intestinal mucosa, which might consequently contribute to the antitumour property of CWQ in CAC disease.

### CWQ ameliorates pro-inflammatory response in CAC mouse model

**Figure 3.**
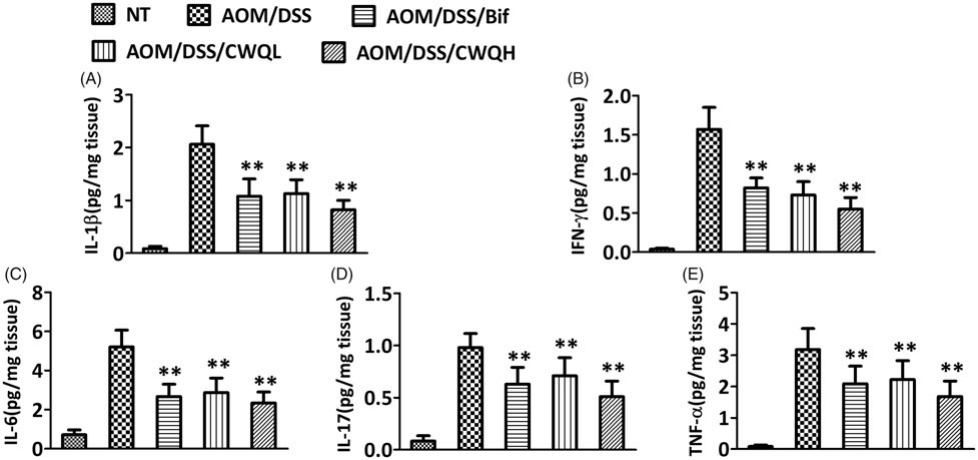
Chang-wei-qing ameliorated pro-inflammatory response in CAC mouse model. Cytokine levels in colonic tissue were measured by ELISA. (A) IL-1β. (B) IFN-γ. (C) IL-6. (D) IL-17. (E) TNF-α. Data are means ± SD (*n* = 6 for each group). Experiments were repeated for three times. **p < 0.01 vs. AOM/DSS group.

Our previous results implicated that both Bif and CWQ could ameliorate colitis in the CAC model mice. Next, we further characterized the pro-inflammatory response to drug treatment in detail. The pro-inflammation cytokines including IL-1β (Figure 3(A)), IFN-γ (Figure 3(B)), IL-6 (Figure 3(C)), IL-17 (Figure 3(D)) and TNF-α (Figure 3(E)) were extracted from colon tissues and measured using the commercially available ELISA kits. We noticed that tremendous pro-inflammatory response was stimulated in the CAC model mice in comparison with the control mice. Consistent with the abovementioned antitumour activity and impact on the microbiota, Bif treatment significantly ameliorated the pro-inflammatory response. Furthermore, high dose of CWQ exhibited comparable effects in resolving inflammatory. Meanwhile, moderate influences were also observed when low dose of CWQ was applied. Therefore, the evident anti-inflammation effects of CWQ were demonstrated, which might convergently contribute to its antitumour activity.

### CWQ decreased TLR4/NF-κB and STAT3 signalling genes in CAC mouse model

**Figure 4.**
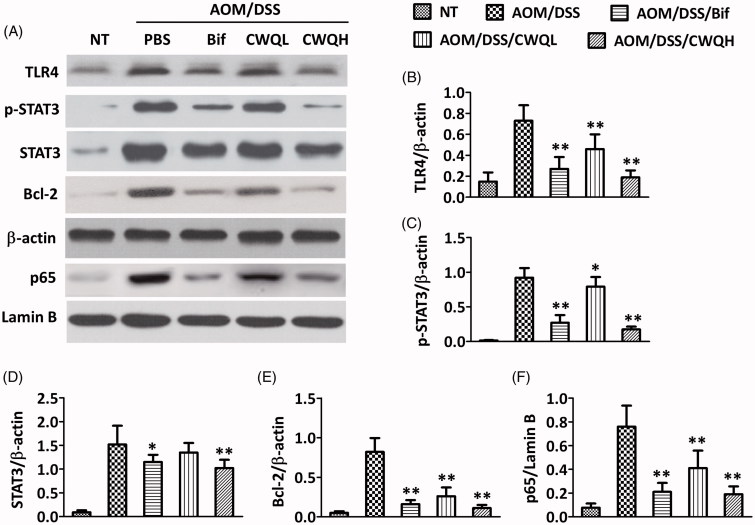
Chang-wei-qing decreased TLR4/NF-κB and STAT3 signalling genes in CAC mouse model. (A) Genes levels in colonic tissue were measured by western blotting. (B–E) Semi-quantitative analysis of TLR4, p-STAT3, STAT3 and Bcl-2 proteins expression compared with β-actin. (F) Semi-quantitative analysis of p65 protein expression compared with lamin B. Data are means ± SD. Experiments were repeated for three times. *p < 0.05, **p < 0.01 vs. AOM/DSS group.

Next, we sought to elucidate the molecular mechanism underlying the anti-inflammation effects of CWQ. Therefore, the critical upstream factors along NF-κb signalling, such as TLR4 and p65, were examined by Western blotting in the colon tissues in response to either Bif or CWQ treatment. The representative immunoblotting images are presented in Figure 4(A), and the corresponding semi-quantitative results are shown in Figure 4(B–F). In line with its anti-inflammatory actions, CWQ treatment significantly decreased TLR4 and p65 proteins to the extent comparable with Bif administration. Slightly inhibitory effects on TLR4/p65 expression were also observed in the low dose CWQ group. Furthermore, we characterized STAT3 signalling in the CAC model mice, which was reportedly associated with colorectal cancer. Both Bif and high dose CWQ remarkably inhibited the activation of STAT3 and expression of Bcl-2, while the total STAT3 protein was slightly decreased. Our data indicated that CWQ ameliorated pro-inflammatory response via suppression of TLR4/NF-κB activation. Meanwhile, the aberrant overactivation of STAT3 signalling in the CAC model mice was greatly abolished by CWQ as well. Our data suggested that CWQ played its antitumour role through both inflammation resolution and STAT3 inhibition.

## Discussion

In this study, we systematically investigated the potential beneficial effects of the Chinese herbal formula, CWQ, on CAC development, and attempted to elucidate the underlying molecular mechanisms in detail. Several essential ingredients of CWQ were found to possess antitumour activities in traditional Chinese medicine. Both *in vitro* and *in vivo* investigations showed *Astragalus membranaceus* with evident immune enhancing effects and anti-inflammation actions. Research demonstrated that *largehead atractylodes rhizome* improved the functions of gastrointestine and suppressed tumour progression. Similarly, *Radix Codonopsis* was often included into the formulas to treat asthma and tumours. CWQ was widely prescribed for the treatment of digestive system malignancies, and impressive clinical efficacy was noticed in suppressing post-surgery recurrence and metastasis, alleviating toxic and side effects of chemotherapies, surmounting drug resistance and prolonging overall survival. To this purpose, we first established the CAC mouse model following the well-established protocol. The successful establishment of CAC was histologically confirmed by colitis score and pathological evaluation. We noticed that 100% incidence of colon tumour was achieved by our method, which was constant regardless of Bif, low dose or high dose CWQ treatments. The tumourigenesis was intimately associated with shortening of total colon length, which was significantly improved by either Bif or high dose CWQ. Consistently, both tumour number and average tumour size were remarkably inhibited in response to high dose CWQ. Our data for the first time demonstrated the comparable antitumour activities of CWQ with Bif in CAC mouse model. Notably, the colitis scoring results suggested that CWQ tremendously ameliorated the inflammatory response in the CAC model mice.

Dysbacteriosis was increasingly implicated in the tumour biology of CACs. Therefore, we further evaluated the alterations of intestinal flora in the CAC mice in response to CWQ treatment. Total bacteria were consistent across normal control, CAC, Bif, low and high dose CWQ groups, while the population of *F. prausnitzii* was markedly enriched in CAC model mice, which was increasingly recognized relating to the tumourigenesis of colorectal cancer. Both Bif and high dose of CWQ greatly suppressed the expansion of *F. prausnitzii*, which might mechanistically contribute to its antitumour properties.

The β-glucuronidase derived from intestinal bacteria was critical in the tumourigenesis of colorectal cancer during a variety of carcinogens exposure. For instance, Summart and Chewonarin (2014) suggested that purple rice extract-supplemented diet reduced dimethylhydrazine (DMH)-induced aberrant crypt foci in the rat colon by inhibition of bacterial β-glucuronidase. Sun and Li (1988) demonstrated the induction of β-glucuronidase activity during DMH carcinogenesis and the additive effects of cholic acid and indole. Cheng et al. (2018) proposed that pharmacological inhibition of β-glucuronidase prevented irinotecan-induced diarrhoea without impairing its antitumour efficacy *in vivo*. Feng and Song (1999) identified the negative correlation of β-glucuronidase with differentiation and invasion of human colorectal carcinoma. Kawee-Ai and Kim (2014) demonstrated that application of microalgal fucoxanthin reduced the colon cancer risk via inhibition against β-glucuronidase in DLD-1 cancer cells. In addition, Waszkiewicz et al. (2015) proposed that serum β-glucuronidase as a potential colon cancer marker based on their preliminary study. In line with the recognized oncogenic properties, here we provided evidences suggesting that β-glucuronidase might serve as the direct target of CWQ and Bif, and suppressed β-glucuronidase activity mechanistically contributed to the antitumour activity of CWQ. In addition, we also demonstrated that both CWQ and Bif greatly improved the intestinal mucosa integrity, which was indicated by the decreased serous levels of d-lactose and endotoxin in the CAC model mice.

The pro-inflammatory response was further characterized in our CAC model mice. Our quantitative analysis demonstrated that remarkable inflammatory reaction was stimulated in colitis animals, which was greatly resolved by administration of either Bif or high dose CWQ. In view of the important roles of chronic inflammation in tumourigenesis of colorectal cancer (Moossavi and Bishehsari 2012), our data indicated that CWQ inhibited tumour progression partially via resolution of inflammatory environment. The molecular mechanism underlying the overactivation of pro-inflammatory response was further attributed to the upregulation of NF-κB signalling.

Accumulative evidence suggested that aberrant activation of STAT3 was intimately associated with the incidence of colorectal cancer (Han and Theiss 2014). Lin et al. (2011) demonstrated that STAT3 was constitutively activated in colon cancer-initiating cells, and established the powerful rationale to develop STAT3 inhibitory strategies for treating advanced colorectal cancers. Spitzner et al. (2014) showed that STAT3-specific siRNA significantly sensitized colorectal cancer to chemoradiotherapy both *in vitro* and *in vivo*. In agreement with this notion, here we provided evidences indicating the over activation of STAT3 signalling in our AOM-induced CAC model. Our data also highlighted that high dose of CWQ significantly inhibited the STAT3 signalling, which convergently contributed to its antitumour activity.

## Conclusions

In summary, our study demonstrates the evident suppressive effects of CWQ against colitis-associated colorectal cancer progression via modulation of microbiota, inflammation resolution and inhibition of STAT3 signalling.
